# Clade-level Spatial Modelling of HPAI H5N1 Dynamics in the Mekong Region Reveals New Patterns and Associations with Agro-Ecological Factors

**DOI:** 10.1038/srep30316

**Published:** 2016-07-25

**Authors:** Jean Artois, Scott H. Newman, Madhur S. Dhingra, Celia Chaiban, Catherine Linard, Giovanni Cattoli, Isabella Monne, Alice Fusaro, Ioannis Xenarios, Robin Engler, Robin Liechti, Dmitri Kuznetsov, Thanh Long Pham, Tung Nguyen, Van Dong Pham, David Castellan, Sophie Von Dobschuetz, Filip Claes, Gwenaëlle Dauphin, Ken Inui, Marius Gilbert

**Affiliations:** 1 Biological Control and Spatial Ecology, Université Libre de Bruxelles, Brussels, Belgium; 2 Emergency Center for Transboundary Animal Diseases (ECTAD), Food and Agriculture Organization of the United Nations, Hanoi, Viet Nam; 3 Department of Animal Husbandry & Dairying, Government of Haryana, India; 4 Earth and Life Institute (ELI), Université catholique de Louvain (UCL), Louvain-la-Neuve, Belgium; 5 Department of Geography, Université de Namur, Namur, Belgium; 6 Joint FAO/IAEA Division of Nuclear Techniques in Food and Agriculture Department of Nuclear Sciences and Applications, International Atomic Energy Agency, Seibersdorf, Austria; 7 Istituto Zooprofilattico Sperimentale delle Venezie, Legnaro (Padua), Italy; 8 Swiss-Prot & Vital-IT group, Swiss Institute of Bioinformatics (SIB), Lausanne, Switzerland; 9 Center for Integrative Genomics (CIG), University of Lausanne, Lausanne, Switzerland; 10 Department of Animal Health, Epidemiology Division, Ministry of Agriculture and Rural Development, Hanoi, Viet Nam; 11 Emergency Center for Transboundary Animal Diseases (ECTAD), FAO Regional Office for Asia and the Pacific (FAO-RAP), Bangkok, Thailand; 12 Animal Production and Health Division (AGAH), Food and Agriculture Organization of the United Nations (FAO), Rome, Italy; 13 Fonds National de la Recherche Scientifique, Brussels, Belgium

## Abstract

The highly pathogenic avian influenza (HPAI) H5N1 virus has been circulating in Asia since 2003 and diversified into several genetic lineages, or clades. Although the spatial distribution of its outbreaks was extensively studied, differences in clades were never previously taken into account. We developed models to quantify associations over time and space between different HPAI H5N1 viruses from clade 1, 2.3.4 and 2.3.2 and agro-ecological factors. We found that the distribution of clades in the Mekong region from 2004 to 2013 was strongly regionalised, defining specific epidemiological zones, or epizones. Clade 1 became entrenched in the Mekong Delta and was not supplanted by newer clades, in association with a relatively higher presence of domestic ducks. In contrast, two new clades were introduced (2.3.4 and 2.3.2) in northern Viet Nam and were associated with higher chicken density and more intensive chicken production systems. We suggest that differences in poultry production systems in these different epizones may explain these associations, along with differences in introduction pressure from neighbouring countries. The different distribution patterns found at the clade level would not be otherwise apparent through analysis treating all outbreaks equally, which requires improved linking of disease outbreak records and genetic sequence data.

The association between geographical distribution of highly pathogenic avian influenza (HPAI) H5N1 subtype and spatial/environmental factors has received considerable attention since the first observation of the virus in China in 1996^1^ and its subsequent spread to Asia, Europe and Africa^2^. The continental-scale spread of the virus raised many economic and health concerns. The introduction of the virus in previously unexposed countries triggered massive efforts to detect and control the unfolding epizootics. In this context, spatial risk mapping helped in identifying areas where surveillance and control should be targeted. Over the period 2004–2014, the virus was introduced in more than 60 countries and in almost all cases, the virus did not persist. However, due to specific conditions in poultry production and value chain systems, and/or limited veterinary resources, the disease has remained endemic or recurrent in China, Viet Nam, Indonesia, Egypt, Bangladesh and Nepal^3^,^4^.

Over its history of invasion, the H5N1 HPAI virus diversified into several genetic lineages that were classified under the nomenclature of “genotypes”. Initially, these genotypes were named after letters or place names. However, with the virus evolving fast and these genotypes multiplying, this system was inadequate and a unified nomenclature based on the phylogeny of the hemagglutinin (HA) gene was established in 2008, when the viruses were classified into clades and subclades^5^. Since 2008, this nomenclature has been revised thrice in 2009, 2012 and 2014^6^,^7^,^8^ to reflect the evolution of the H5 hemagglutinin (HA) gene. To quickly identify clades, determination tools relying on genetic databases were developed based on the updated nomenclature^9^. Over the years, some clades only appeared briefly and quickly disappeared (e.g. clade 9), others (or their descendants) persisted over very long periods of time (e.g. clade 1), and some clades had a very successful temporary history, spreading to many countries before being superseded by another (e.g. clade 2.3.4). The geographical distribution of HPAI H5N1 clades has specificities. For example Egypt has only been infected with 2.2 and derived clades. Indonesia had only been infected with 2.1 and derived clades, until the introduction of 2.3.2 clade was reported in 2012^10^. China has the highest diversity of clades.

Previous studies on the geographical distribution of HPAI H5N1 have focussed on agro-ecological conditions associated with the presence of the H5N1 subtype without making any distinctions among virus clades^2^. In parallel, descriptive studies focussed on the spatio-temporal distribution of different clades^3^, but to date, no clade-level statistical analysis of HPAI H5N1 in relation to spatial risk factors has been performed, a gap we intend to fill.

HPAI H5N1 cases have been reported worldwide but there is evidence of local persistence of the virus in only some countries in Asia and Africa^11^. Since 2003, the Mekong region has been affected by several epidemic waves involving numerous circulating clades of HPAI H5N1. In Viet Nam, the circulation of several clades in poultry population is well documented: H5N1 HPAI was first identified in poultry in 2001^12^ and the most frequent clades of HPAI H5N1 viruses sampled in the country belong to clades 1, 2.3.2, 2.3.4, and their descendants. Clade 1 was introduced in 2003 in Viet Nam and viruses from the clade 1 monophyletic tree remained dominant in the Mekong delta^12^. From 2005 to 2009, the clade 2.3.4 viruses superseded clade 1 viruses in northern Viet Nam and were linked to the majority of outbreaks during that period^13^. Clade 2.3.2 circulated briefly in the northern region during 2005–07, and again made a reappearance in 2009, eventually superseding clade 2.3.4 in the northern region^14^.

The spatial distribution of these clades in the country was never studied quantitatively, because of a lack of comprehensive data sets linking HPAI H5N1 sequences and outbreak locations. So, on one hand, databases on HPAI H5N1 outbreaks contain records with good spatio-temporal accuracy, but no information on clade or other virus features. On the other hand, HPAI H5N1 sequence databases with information on clade, are only identified with relatively coarse descriptive information on the location and time when the sequence was sampled. The absence of an explicit link between those two types of databases has, for many years, prevented spatially-detailed analyses of clade-level outbreak data. To try and fill these gaps, the Food and Agriculture Organisation of the United Nations (FAO) developed a genetic module within its epidemiological platform, the FAO Emergency Prevention System Global Animal Disease Information System (EMPRES-i^15^), linking influenza events contained in EMPRES-i with influenza sequences contained in OpenFlu^16^. To date however, only a fraction of HPAI H5N1 outbreaks contained in EMPRES-i have been explicitly linked to an existing virus sequence.

In this study, we compiled a large set of HPAI H5N1 outbreaks and virus sequences for the Mekong region including Viet Nam, Cambodia, Laos and Thailand. HPAI H5N1 outbreaks were then linked to sequences with a probabilistic procedure to reconstruct a time series of outbreaks tagged with clade information. The clade assignment of individual sequences or isolates was derived from phylogenetic analysis and used under the assumption that these would represent homogeneous sub-populations of HPAI H5N1 viruses. The objective of the present study was to assess the association between different clades and agro-ecological factors in space and time with the use of Boosted Regression Tree (BRT) spatial suitability models with bootstrapping to account for uncertainty in the linkage between outbreaks and sequences.

## Results

**Figure 1:**
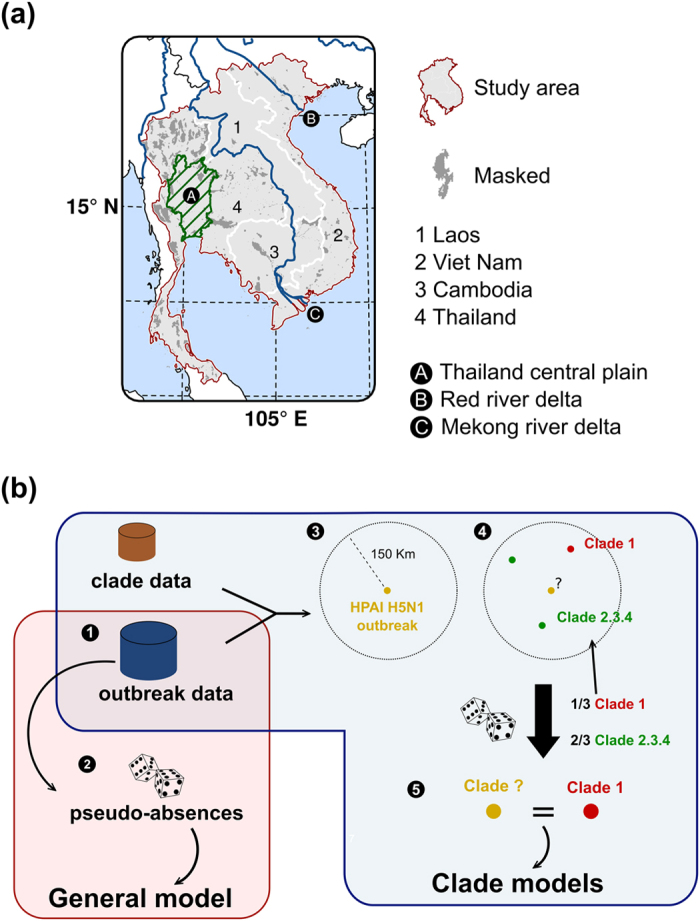
Descriptive information. **(a**) Study area. Three localities were marked on this map: A: The central plain of Thailand (the green hatched area); B: The Mekong Delta; C: The Red river Delta. The dark grey areas show the mask used in this study and corresponds to International Union for Conservation of Nature (IUCN) conservation areas, permanent water bodies and city centres where poultry is presumable absent. The graticule is composed of a 5-degree increments (http://www.naturalearthdata.com/) and the coordinate system is ‘EPSG:3148’. The data used to produce these maps were all from public sources, and the country limit data are from the FAO Global Administrative Unit Layers (GAUL) database. This figure was built with the R-3.2.3 software (https://cran.r-project.org/). **(b**) Summary diagram of the models and outbreaks clade allocation steps. b. 1. General model (red box): Step 1: Extraction of oubreak dataset (EMPRES-i (http://empres-i.fao.org/); Department of Animal Health (Hanoi, Viet Nam)); Step 2: Random sampling of pseudo-absences from the population at risk and computation of ‘P(H5N1)’, the HPAI H5N1 presence probability. The random sampling procedure of pseudo-absences is repeated for each bootstrap of the analysis. b. 2. Clade models (blue box): procedure for linking outbreaks to HPAI H5N1 sequences and the clade models. Step 3: A buffer of a given distance is drawn around each outbreak location (outbreak data) and the sequence data were extracted (Bioinformatics OpenFlu database (http://openflu.vital-it.ch/); Influenza Research Database (IRD, http://www.fludb.org/)); Step 4: Sequences located within that buffer are identified. Step 5: One of these sequences is randomly selected and used for analysis. The entire allocation procedure is repeated for all outbreaks and bootstraps of the analysis.

From January 2004 to November 2013, 6,823 HPAI H5N1 outbreaks and 1,485 sequences with locations were recorded. The clade assignment procedure (Fig. 1) allowed assigning a clade to 6,490 outbreaks (95% of outbreaks; Supplementary Table S1). The uncertainty in the assignment procedure was reasonable, with a median of 1 and a mean of 1.45 distinct clades/sequences to choose from per outbreak. This means that in the majority of cases, there were few candidate clades to choose from within the space-time assignment window of each outbreak. Lower or higher distance threshold in the space-time windows resulted in either >5% of outbreaks with missing clade, or with a higher mean number of clade/sequence to choose from per outbreak (Supplementary Text S2).

**Figure 2:**
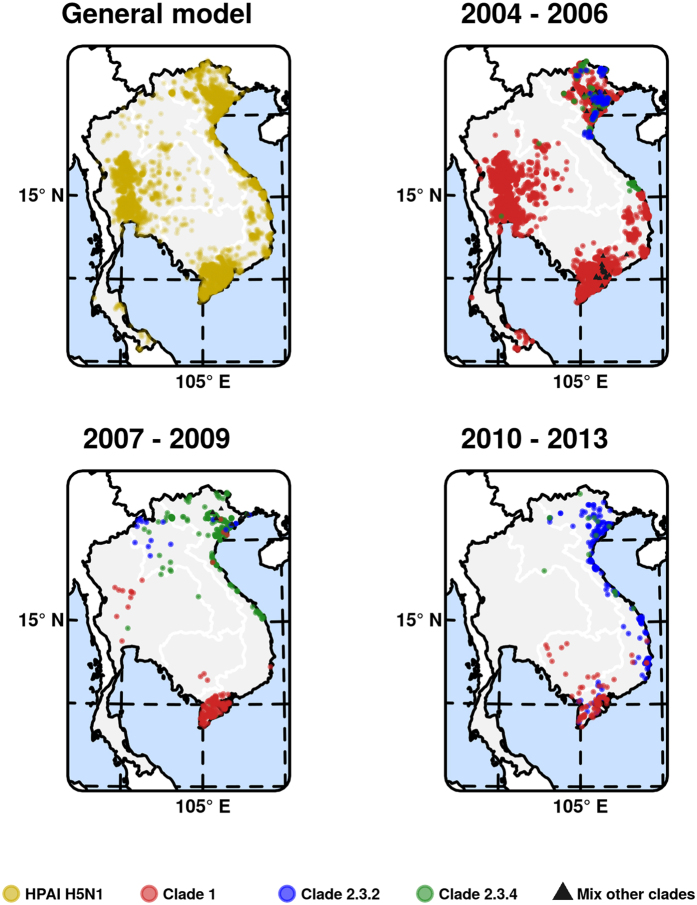
Distribution maps of outbreaks. Distribution of outbreaks (yellow) used for the HPAI H5N1 general model (top left), and maps of one realization of the outbreaks with clade assignation for the three time periods. Note that the colours of points individual points is transparent such as to show areas with overlapping points with more saturated colours. The mask corresponds to International Union for Conservation of Nature (IUCN) conservation areas, permanent water bodies and city centres where poultry is presumable absent. The graticule is composed of a 5-degree increments (http://www.naturalearthdata.com/) and the coordinate system is ‘EPSG:3148’. The data used to produce these maps were all from public sources, and the country limit data are from the FAO Global Administrative Unit Layers (GAUL) database. This figure was built with the R-3.2.3 software (https://cran.r-project.org/).

The distribution of outbreaks and sequences was very unbalanced per country and time-period. Figure 2 and Table 1 show the distribution and counts of sequences and outbreaks after their clade assignment, for the three considered periods. The large majority of outbreaks occurred within the first time-period of 2004–2006 in Viet Nam and Thailand (n = 6103, Fig. 2), whereas the periods 2007–2009 and 2010–2013 had decreasing numbers of outbreaks, with 439 and 169 outbreaks respectively.

**Figure 3:**
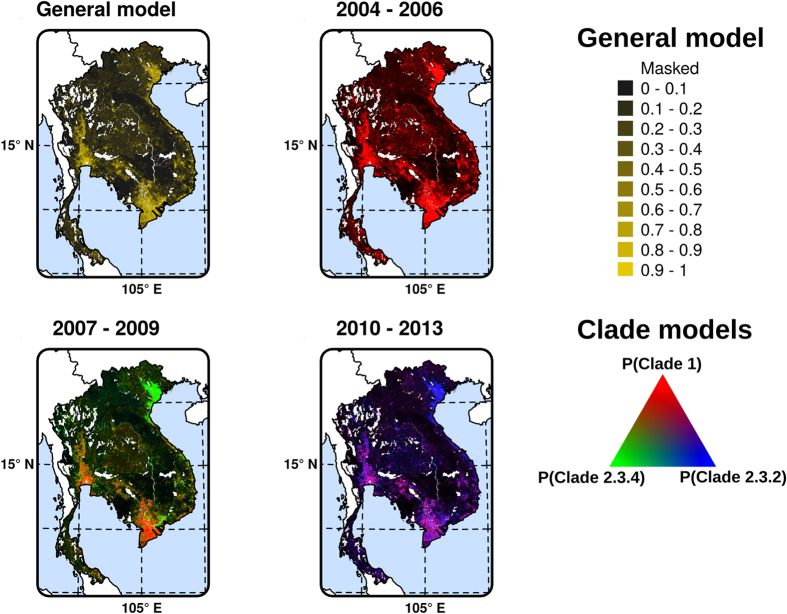
Suitability model predictions. General HPAI H5N1 suitability model (P(HPAI)) for the entire time period (**a**); and for each clade P(Clade) in their respective time periods (**b**), with colour intensity proportional to the predicted suitability for a particular clade. Note that in Red, Green, Blue (RGB) visualisation, pixels in black correspond to pixels where the predicted suitability for all three clades was close to zero, as the intensity of the colour varies with suitability. The mask corresponds to conservation areas of International Union for Conservation of Nature (IUCN), permanent water bodies and city centres where poultry is presumable absent. The graticule is composed of a 5-degree increments (http://www.naturalearthdata.com/) and the coordinate system is ‘EPSG:3148’. The data used to produce these maps were all from public sources, and the country limit data are from the FAO Global Administrative Unit Layers (GAUL) database. This figure was built with the R-3.2.3 software (https://cran.r-project.org/).

The general HPAI H5N1 model, i.e. contrasting HPAI H5N1 outbreak presence points with pseudo-absences points had a good accuracy, with a mean area under curve (AUC) of 0.825 ± 0.009 (mean ± standard deviation), estimated on the validation set, and averaged over different sets of pseudo-absences. The general suitability map (Fig. 3) highlights areas of high suitability for HPAI H5N1 infections, and strongly resembles the output produced by auto-logistic regression models of the first three waves of infection mapped in Gilbert *et al*.^17^. A high suitability for HPAI outbreaks is found in Thailand central plain, in the Red river and Mekong river Deltas of Viet Nam, and in parts of Cambodia along the Mekong river system (Fig. 1).

**Figure 4:**
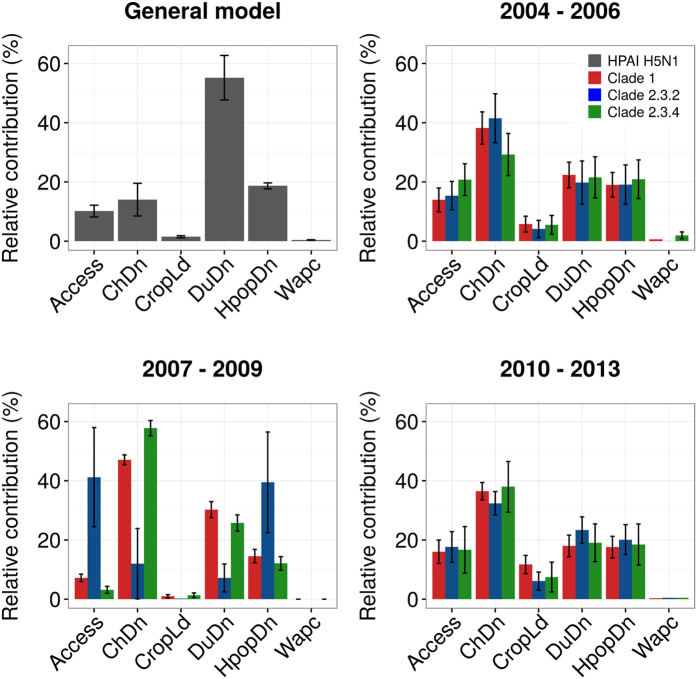
Mean relative contribution of each predictor variable to the prediction for the general and clade models for individual time periods. The relative contribution plots show the statistic importance of each predictor variable in the model. It is measured as a percentage on the Y-axis for each predictor variable: human population density (HpopDn, human/km^2^ - log10), chicken density (ChDn, head/km^2^ - log10), duck density (DuDn, head/km^2^ - log10), cropping intensity (CropLd, intensity scale from 0 to 4), accessibility (travel-time) to major cities (Access, min), and the proportion of land occupied by water (Wapc). Log10 notes a logarithm transformation of base 10 of predictor variables.

**Figure 5:**
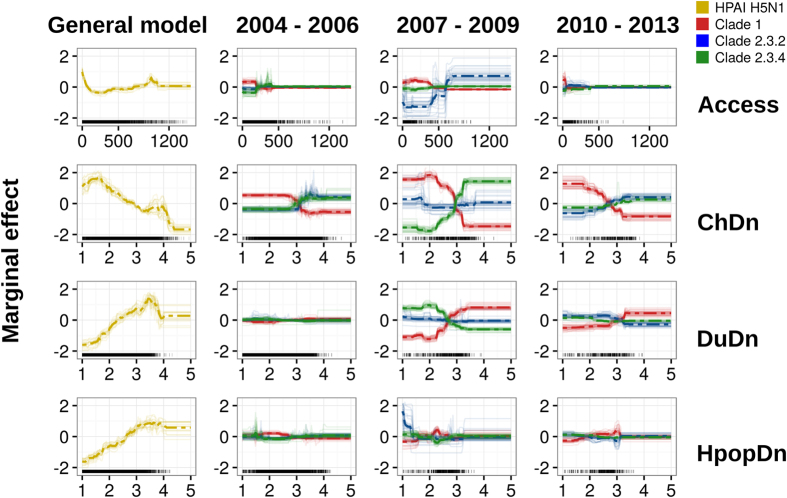
Partial dependence plots or BRT profiles for the four top predictor variables for the general suitability model and for individual clade models. The partial dependence plots show the predicted dependence between the dependant variable of BRT models on the X-axis (the probability of virus presence) and each predictor variable on the Y-axis. Four predictor variables were selected for this figure: human population density (HpopDn, human/km^2^ - log10), chicken density (ChDn, head/km^2^ - log10), duck density (DuDn, head/km^2^ - Log10) and accessibility (travel-time in minute) to major cities (Access, min). Log10 notes a logarithm transformation of base 10 of predictor variables. The dashed line represents the mean profile, whilst transparent lines represent each bootstrap. The black transparent ticks at the bottom of each plot represent the observed distribution of predictor variables for one bootstrap and the corresponding dataset.

The clade-level models, i.e. contrasting points of a particular clade against HPAI H5N1 points with other clades for a given period of time, had moderate to good predictive accuracy, with AUC ranging from 0.82 to 0.98 when evaluated against the model calibration data set, and from 0.68 to 0.90 when evaluated with the cross-validation implemented by Elith *et al*.^18^ (Table 2). The relative contribution of the different predictor variables and the dependency profiles of the predicted values are presented in Figs 4 and 5 respectively. Chicken density, human population density and duck density were the spatial factors with the highest relative contribution to the different clades and time periods. These factors were followed by accessibility (travel time) to major cities, the amount of cropland, and the proportion of water, which only marginally contributed to the different models. The most interesting observation can be made from the dependency profiles for the four most important variables (Fig. 5). In interpreting those profiles, one should remember that clade 1 was largely dominant in period I, and that clades 2.3.4 and 2.3.2 were most abundant in period II and III, respectively. Our results show that clade 1 (Fig. 5, red) is always statistically associated with low chicken density and low travel time to important cities. This contrast with clades 2.3.2 and 2.3.4, which are found to be statistically associated with high chicken density in period I, period II (particularly 2.3.4) and period III. A similarly contrasting pattern, though inversed, can be observed with clade 1 being statistically associated with high duck density in period II and period III. These relationships are well captured in the Red Green Blue (RGB) composite plots in Fig. 3 that highlight the widespread dominance of clade 1 in period I, the coexistence of clade 1 and clade 2.3.4 in period II, and of clade 1 and clade 2.3.2 in period III. The Mekong Delta seems in all cases to be a particular area, where clade 1 succeeded in persisting during the entire study period, being challenged by clade 2.3.4 during period II, and by clade 2.3.2 in period III. But it is noteworthy that the two latter clades never apparently succeeded in superseding clade 1 in the Mekong Delta. In contrast, the Red river Delta, shows a much more clear-cut succession of dominant clades 1, 2.3.4 and 2.3.2 with very limited temporal co-existence of clades.

## Discussion

The three areas of high suitability for HPAI H5N1 infection that we identified are in agreement with previously published results on HPAI H5N1 risk mapped for Asia^19^, Thailand^17^,^20^,^21^ or Vietnam^17^,^22^. For example, they strongly overlap the output produced by auto-logistic regression models of the first three waves of infection mapped in Gilbert *et al*.^17^. They are the Thailand central plain, the Red River and Mekong Deltas of Viet Nam, and parts of Cambodia along the Mekong river system, along with a limited number of areas of suitability in Laos. These regions correspond to the main poultry production areas, where irrigated landscapes show a very dense mix of people, chicken and duck production and cropland^17^,^21^. The spatial overlap with previously published risk maps is not surprising as many predictor variables and outbreak location data were common, the main difference being the method that was employed (BRT vs. autologistic regression models) and a few differences in predictor variables (elevation was not considered here and the proportion of land covered by water was not considered in Gilbert *et al*.^17^). Within the high suitability area, a clear spatio-temporal pattern of clade distribution emerges from our analyses. Clade 1 dominated all landscapes during 2004–2006, was then replaced in the Red River Delta by clade 2.3.4 in the 2007–2009 period and by clade 2.3.2 in the 2010–2013 period. In contrast, clade 1 persisted in the Mekong Delta, despite clades 2.3.4 and 2.3.2 being introduced. Interestingly, the persistence of clade 1 in the Mekong Delta is correlated with a high duck density, whilst the more novel clades 2.3.4 and 2.3.2 correlated more strongly with high chicken and human population densities, as found in northern Viet Nam’s Red River Delta.

Discussing these results is best done in the broader context of the spatio-temporal epidemiology of HPAI H5N1 in Asia. Clade 1 was first reported in southern China in 2002, in a region that appears as an important source of HPAI H5N1 diversity^23^. The virus then likely spread to Viet Nam from the Yunnan region of China through transboundary poultry trade in the common value chain between the two countries^24^, especially in light of the cultural preference to use Chinese (instead of Viet Namese) spent hens for making local soup (Pho) in northern Viet Nam^25^. Clade 1 was also the dominant clade in Thailand, Laos, Cambodia and Viet Nam during the 2004–2006 epidemic waves^3^ and became a major public health and economic concern. In response, all countries in the region tried to eradicate HPAI H5N1 using a combination of preventive and control measures including stamping out, vaccination, movement control, cleaning and disinfection. In Viet Nam, eradication of HPAI H5N1 was attempted from December 2003 through March 2004 by culling 45 million poultry^25^ and in 2005 through restriction of animal movement^22^. The combined effects of the government measures may have aided in preventing further spread of infection to the southern and central parts of Viet Nam in 2005/2006. It may have also played a role in limiting clade 1 to the central and northern parts of Viet Nam. Thailand’s government also acted very strongly against HPAI H5N1 through the implementation of massive, bi-annual, large-scale active surveillance campaigns involving several hundred thousand volunteers, which contributed to a very detailed census and early detection of outbreaks. One may note that the country never used vaccination as part of its prevention and control strategy.

In period II (2007–2009), the spread of HPAI H5N1 was more limited and viruses belonging to clade 1 became entrenched in the Mekong region of southern Viet Nam. Just prior to this period, the new HPAI H5N1 clade 2.3.4 emerged in China in 2005 and became dominant in southern China during period II, from 2007 to 2009^13^,^26^.

Mass vaccination of poultry for avian influenza became compulsory in China and began in 2004 with the vaccine strain Re-1 derived from the virus A/goose/Guangdong/1/1996^27^. It has been hypothesized that this first vaccination campaign could have contributed to the emergence and spread of clade 2.3.4 by one of three hypothesized mechanisms: i) poultry vaccinated against HPAI H5N1 having poor sero-conversion rates leading to the emergence of clade 2.3.4 vaccine-escape variants^26^ as observed in China for H5N2 virus^28^; ii) antigenic drift in sub-lineage HPAI H5N1 variants may have reduced vaccine efficacy in poultry^29^ leading to clade 2.3.4 emergence; or iii) inadequate vaccination coverage in waterfowl leading to infection and subsequent viral evolution and emergence in ducks^30^. Emergence and establishment in southern China may also be a result of a combination of these factors, but the spread into Viet Nam is most likely a result of unofficial or illegal poultry movement pathways. This could have been amplified by the sequence of events. Nearly 17% of Viet Nam national poultry population was culled, mostly in the north, in an attempt to control the disease in 2004, and this caused a strong shock to the market that forced many small local operators out of production. The local demand for poultry may likely have been very high temporarily, as local production could not meet the high demand in the highly populated Red River Delta region including Hanoi^25^.

Interestingly, clade 2.3.4 was confined mainly to the north and east-coast of Viet Nam, and to Laos, but never invaded the Mekong Delta in the 2007–2009 period. A similar observation can be made about Clade 2.3.2 during the period 2010–2013, which emerged outside the Mekong Region^31^, and was later found in Viet Nam during several separate outbreak events^14^. Clade 2.3.2 quickly replaced clade 2.3.4 viruses in north and central of Viet Nam, but never succeeded to invade southern Viet Nam. The emergence and rapid spread of clade 2.3.2 may be the result, once again, of the selection of a new vaccine-escape variant. Indeed, in 2008, the Re-1 vaccine was replaced in poultry production of China by a new vaccine from A/duck/Anhui/1/06 (clade 2.3) and was designated as Re-5^27^.

Hence, this depicts two particular epidemiological zones (epizones) for HPAI spread and persistence in the region, one associating southern China to northern Viet Nam with a succession of clades replacing each others, and the other associating the Mekong Delta and Cambodia with long-term persistence of clade 1 and subsequent additional evolution in Cambodia^32^. The Mekong and Red River deltas differ in many aspects, but from a poultry production system perspective, the main difference is the presence of more intensified chicken production systems in the north Red River Delta, and higher relative densities of ducks in the south Mekong River Delta. Flock size also differs, with generally higher number of birds per owner in the north, translating into higher densities of chicken per km^2^. Accordingly, in our BRT models, we found chicken and duck densities to be the one best available to discriminate the suitability for clade 1 on the one hand, and clade 2.3.4 and 2.3.2 viruses on the other. Therefore, two main hypotheses could be drawn to explain the different patterns of associations between these clades and poultry variables.

First, if we assume that viruses from all clades have an equivalent fitness and patterns of excretion and transmission, this could be the result of differences in surveillance, prevention and control in these different areas, combined with novel introduction from China in the North. Domestic ducks have always been strongly associated with areas of persistence and evolution of H5N1 HPAI^33^, which has been demonstrated in both host-pathogenicity^34^ and geospatial studies^17^. A comparatively higher density of domestic ducks in the Mekong delta may hence better favour long-term persistence compared to northern Viet Nam, leaving fewer opportunities for the introduction of novel clades. In contrast, lower densities of ducks in northern Viet Nam could have limited the persistence potential of clade 1 through the domestic duck reservoir, reducing its circulation, hence leaving more opportunities for introduction of new clades from China. Furthermore, the intensive poultry production systems of the north have a high turnover of poultry as compared to backyard and smallholders commercial duck farms. This provides a stock of immunologically naive hosts that are more susceptible to novel clades than ducks. Many factors that would differentially affect chicken and duck poultry production systems, such as shared vaccines or vaccinators, shared stockbreeders, involvement of similar product collectors, or participation to similar market networks, would be comparatively more important in the northern part of the country, and may explain some of the observed differences in persistence. In addition, whilst vaccines for clade 2.3.4 and 2.3.2 were apparently imported from China to supply Viet Nam, access to vaccines against clade 1 viruses was difficult due to the lack of production in China, and by the difficulty in scaling up production within Viet Nam. Finally, some factors may have prevented the spread of clades 2.3.4 and 2.3.2 further south. For example, in contrast to the north, large populations of poultry in southern Viet Nam were not destroyed in an attempt to control the disease. Viet Nam started restricting animal movement between provinces, which may have limited the introduction of clade 2.3.4 and 2.3.2 into southern Viet Nam as local production in the south was sufficient to meet the demand. From this perspective, it is likely that circulation of clade 2.3.4 and 2.3.2 remained particularly limited. This is supported by the study of Minh *et al*.^35^, showing that local spread of virus was more predominant than long-distance spread during the 2009 epidemic in the Mekong river Delta, with household-to-household infection rate within communes being in the order of 50 times greater than the household-to-household infection rate between communes.

Second, a more speculative hypothesis is that the fitness of clade 1, 2.3.4 and 2.3.2 may differ. For example, clade 1 may have the best fitness to the host composition and poultry production systems found in the south Mekong Delta, which could explain why clade 2.3.4 or 2.3.2 never superseded clade 1, although they were introduced several times. Experimental infection studies have indeed shown that the pathobiology and virulence of a same sub-lineage of HPAI H5N1 could vary significantly among avian species^36^,^37^,^38^, and these features may vary not only between hosts, but also between different virus clades^26^,^39^. Based on the very limited number of experimental infection studies, no strong evidence has been found of a strong difference in pathogenicity between clades 1, 2.3.2 and 2.3.4 HPAI H5N1 viruses, but the hypothesis cannot be entirely ruled out. For instance, duck mortality was reported to be higher due to Clade 2.3.2.1 in ducks from Indonesia^10^.

Although the proportion of detected viruses that are sequenced has significantly increased between 2004 and today, an important limitation of this study remains the fairly poor quality of the georeferencing of HPAI H5N1 sequences, and the spatial heterogeneity in their sampling. We tried to account for this sampling bias through the implemented bootstrapping procedure, and the pattern observed in this appears quite robust to even important change in the definition of the space-time windows. However, this type of validation remains internal, and in the absence of alternative, one can hardly make a detailed assessment of the effect of the sampling bias on our model parameters. In addition, we are confident that our main results contrasting the epizones of Cambodia and the Mekong delta in one hand, with the Red river delta and surrounding area in the other hand, could hardly be compromised by the sampling bias, as there was a sufficiently high number of sequences sampled in each of the area per time period to capture the relative proportion of the different clades studied here. Many of the location data associated to sequences used in this study had to be curated for improvement, by returning to the original reference to infer the most likely location of the sampling. This calls for improvement of georeferencing accuracy in public sequence repositories such as Genbank, and for more widespread use of linkages between sequence and outbreak data, which extends to all diseases beyond the specific case of avian influenza considered in this study. A first attempt to record validated linkages between outbreak data and sequences was done in the EMPRES-i genetic module. But this could be part of a more formal recommendation upon sequence submission as the lack of good location data represents one of the main obstacles to linking phylogeographic information to environmental and risk factors in the molecular epidemiology of avian influenza and many other important diseases. For example, to date, only a few hundreds of the thousands of HPAI H5N1 outbreaks included in the EMPRES-i database have been linked to a sequence through the genetic module. More specifically, as of October 2015, 843 database links between avian influenza records from the EMPRES-i database and isolates from the Openflu database have been validated, including 387 for H5N1 HPAI. The closer integration, i.e. use of common fields and formal linkages, between the EMPRES-i, OpenFlu and IRD databases is recent and was established as a mean to cross-check and improve EMPRES-i epidemiological records. Further developments in the direction of a closer integration will be of interest for future studies, and could be helped by better spatial and temporal accuracy of the sequences metadata.

Another potential limitation of the study is that all the predictor variables tested in the analysis were static in time, so we were unable to capture, for example, the effect of intra-annual (e.g. seasonality) and inter-annual (e.g. intensification of the production) changes in poultry production. Agricultural censuses represent huge data collection efforts and can hardly be repeated regularly. However, preliminary analysis of Thailand x-ray annual survey data revealed relatively low levels of spatial changes (unpublished data), and seasonality of production could be inferred at coarser spatial scale. Future work looking more specifically at temporal patterns of poultry production should be able to better understand the level intra-annual and inter-annual variations in poultry populations.

Our results highlight the value of integrating virus characteristics in spatial models to better understand the emergence and spread of influenza A viruses. Other virus characteristics could potentially have been used from progenitor viruses, however this study focussed on clades, which only reflect evolutionary patterns of one gene segment out of 8. This was justified by the good description of the nomenclature, its standardization, and by the fact that this H5 gene is strongly exposed to selective pressure by the host immune system and it is the only gene derived from the common A/Goose/Guangdong/1/96 ancestor among HPAI H5N1 viruses. However, other virus features would also be of interest for future studies, such as specific mutations (e.g. associated with higher risks to human transmission), evolutionary patterns of other segments such as NA (neuraminidase) and internal genes, and above all, multiple reassortments. Influenza reassortment can be responsible for major genetic shifts of influenza A viruses, as shown with the emergence of the pandemic H1N1 strain in 2009 and of the H7N9 strain in China in 2013. There are still many technical obstacles to the monitoring and description of reassortment events, but whole genome sequencing of influenza A viruses, which is becoming more and more common today, will add significantly to knowledge in this field, and would allow further understanding of risk factors for a future reassorted pandemic strain to emerge and spread.

## Methods

### Outbreak and clade records data

HPAI H5N1 outbreak data in the Mekong region (including Thailand, Laos, Cambodia and Viet Nam) were extracted for the period 1st January 2004 to 13^th^ December 2014 from the database of the Global Animal Health Information System of the FAO (EMPRES-i: http://empres-i.fao.org/). This data set of 6762 outbreak locations was complemented by 338 outbreak location data provided by the Department of Animal Health (Hanoi, Viet Nam). Ninety-eight (98%) of these outbreaks were geo-referenced at the administrative level 3 (commune), 0.5% at the administrative level 2 (district) and 1.5% at the administrative level 1 (province). The definition of an outbreak may have varied over time and by country, but in most cases outbreaks are assumed to represent a farm, or a group of farms where HPAI H5N1 were observed at least once at a point in time.

Two databases were used to obtain information on the spatio-temporal distribution of HPAI H5N1 clades in the same spatio-temporal window: the Swiss Institute of Bioinformatics OpenFlu database (http://openflu.vital-it.ch/), and the Influenza Research Database (IRD, http://www.fludb.org/). Together, these two sources yielded a set of 642 locations with a given clade number for an HPAI H5N1 sequence obtained following outbreak investigation or obtained through active surveillance. In addition, a set of 843 sequence locations, with their respective clade number was provided by the Department of Animal Health (Hanoi, Viet Nam). A large proportion of these sequences with clade information included poor geo-referencing data, typically in administrative level 1. They were therefore curated to improve the quality of the georeferencing, either by searching the paper linked to the reference for more accurate location information, or by directly contacting the authors.

### Assignment of clades to outbreaks

A bootstrapped procedure was implemented to assign clades to outbreaks. For each outbreak, a space-time window was defined with a distance of 150 km, and a temporal window of one year prior to the outbreak date. The distance was chosen as a trade-off to ensure that >95% of outbreaks had at least one sequence within the space-time windows, and yet to do not have a too high number of sequences to choose from for each outbreak. The sensitivity of the model to the parameters of the space windows was tested by repeating the analysis with larger and smaller space-time windows (Supplementary Text S1). One sequence was then randomly sampled from the set of sequences found within the space-time window (Fig. 1), and the clade associated to the randomly sampled sequence was assigned to the outbreak. This was repeated for all outbreaks. The initial data sets included outbreak time and locations for clades 0, 1, 2.3.2, 2.3.4, 5 and 7. All these clades were included in the linking procedure but only clades 1, 2.3.2 and 2.3.4 were included in the final analysis, since these were the only ones with sufficiently high number of outbreaks to allow a spatio-temporal analysis to be conducted over the time period. Eventually, by the end of this process, all outbreaks included in the analysis were assigned to a clade, picked at random within the set of sequence locations found within the space-time windows. This random selection procedure, and its repetition over 30 bootstraps, allowed to account for possible co-circulation of several clades in the same area (e.g. different clades could be assigned to the same outbreak through the repetition of the allocation procedure over different bootstraps), and to ensure that the obtained results were not influenced by one specific allocation.

### Environmental data

We selected the spatial agro-ecological factors (hereafter referred to as “predictors”) used to model the suitability for HPAI H5N1 outbreaks based on a recent review of HPAI H5N1 spatial models^2^. The following six predictor variables were thus obtained at a spatial resolution of 1 km^2^: human population density (HpopDn), chicken density (ChDn), duck density (DuDn), cropping intensity (CropLd), accessibility (travel-time) to major cities (Access), and the proportion of land occupied by water (Wapc). Human population density for the year 2010 data was obtained from the WorldPop project: http://www.worldpop.org/40,41, whilst travel time to major cities was extracted from the global accessibility map of Nelson^42^. The accessibility map was published in 2009 but is derived from data sets with different time points. Poultry density data was obtained from the Gridded Livestock of the World (GLW) database v2.1: http://www.livestock.geo-wiki.org/43 and included census data from 2010, 2009, 2011 and 2012 for Thailand, Laos, Cambodia and Viet Nam, respectively. We also used the GLW mask that excludes conservation areas of International Union for Conservation of Nature (IUCN), permanent water and city centres and where poultry are presumably absent. Data on average cropping intensity were derived from annual maps of cropping intensity and paddy rice agriculture, at 500-m resolution using 2005 satellite images from NASA’s Moderate Resolution Imaging Spectroradiometer^44^. The proportion of land occupied by permanent water was estimated from the Globcover 2009 database^45^. The same predictor variables were employed for the entire study period, i.e. we did not account for possible changes in the spatial distribution of people, poultry or other predictor variables over time.

### Farm density modelling

In addition to the chicken and duck density layers, we also aimed to produce a layer describing the epidemiological units, i.e. the number of farms per pixel, so that it could be used to spatialize presence and pseudo-absence points in a more realistic way than by random allocation. The methods used to construct the poultry farms layer is described as supporting information (Supplementary Text S2).

### HPAI H5N1 outbreak suitability modelling – general model

The suitability maps for HPAI H5N1 outbreaks were generated in a two-step process. All outbreak suitability models described hereafter were calibrated using BRT. First, a BRT model was calibrated using our six predictor variables and all HPAI H5N1 records (all clades pooled together) pooled over the entire study period (2004–2014) as presence records. HPAI H5N1 outbreaks were recorded at a given administrative level and hence, there was a certain level of uncertainty regarding the exact location of the outbreaks. In order to prevent any systematic bias, within each administrative polygon where an outbreak was recorded, a point was randomly sampled from the distribution of chicken and duck farms. Since BRT model calibration requires absence records in addition to presences, we generated pseudo-absences from the distribution of chicken and duck farms, following the pseudo-absence distribution algorithm presented in Pigott *et al*.^46^ accounting for spatial variation of the population at risk, and upon the conditions that they could not fall within the same smallest administrative unit as a presence record. Pseudo-absences were generated in greater numbers than HPAI outbreaks (double of HPAI H5N1 outbreaks) as a trade-off between representing enough variability in the pseudo-absence points and yet keep the prevalence high enough to not suffer from possible bias linked to artificially prevalence^47^.

### HPAI H5N1 outbreak suitability modelling – clade models

Second, clade-level suitability models for clade 1, 2.3.2 and 2.3.4 were built for three different time periods, using the set of outbreaks tagged with a candidate clade. Analyses were grouped in three time periods in order to avail enough data points for building individual models per study period and corresponding to different waves of infection and appearance or disappearance of particular clades: 2004–2006 (corresponding to the first and second waves of infection in Thailand and Viet Nam); 2007–2009 (localised persistence in Thailand, and Viet Nam) and 2010–2013. One should note that the most recent HPAI H5N1 positive case in Thailand was reported in 2008. Thailand is nevertheless maintained in the predictive map, as the prediction map merely represents suitability for infection rather than actual probability of presence. In each model, presence records used for calibration were the outbreaks locations tagged with a particular clade, and absence points were HPAI H5N1 outbreak locations tagged with a different clade. For instance, the model for clade 2.3.2 in the period 2007–2009 was calibrated using all outbreak locations during that time period, with those tagged as belonging to clade 2.3.2 being the presence points, and outbreaks tagged with other clades being used as absence point. The same six predictor variables previously described were used as explanatory variables. The final prediction for a given clade and a given time period, consisted in the combined probability of the general HPAI H5N1 model, and of the particular period and clade model. In summary, the general model can be considered as contrasting the suitability of pixels for HPAI H5N1 infection against the rest of the landscape in general, i.e. P(HPAI H5N1), and each individual clade model contrasts the suitability of a particular pixel for a particular clade against other clades, i.e. P (clade|HPAI). The final prediction for a given clade and a given period, consisted in the combined probability of the general HPAI H5N1 model, and of the particular period and clade model, i.e. the product of P (HPAI H5N1) by P (clade|HPAI).

### Calibration and evaluation of Boosted Regression Tree models

Boosted Regression Tree models (BRT) were used in this study for their ability to account for non-linearity and complex relationships between the dependent variable and the predictor variables^18^. In addition, BRT models were shown to generally outperform more conventional approaches, such as multiple logistic regression, in general species distribution modelling studies^48^, as well as in HPAI H5N1 risk modelling^21^,^49^. The K-fold cross validation procedure from Elith *et al*.^18^ was used to select the optimal number of trees in the model and to check for overfitting. Four sets of training and validation points were subsampled from the full data for cross-validation, a tree complexity of 4, a learning rate of 0.01 and a bag fraction of 75% were also used as parameter values in the calibration of all models. The predictive power of all models was evaluated through two metrics: the area under the ROC curve (AUC), computed for the binary classifier, and the Pearson correlation between the dependant variable and the prediction (Corr).

Many steps along the modelling procedure involved the use of random selection, such as i) the location of presence points within the administrative unit where they were recorded, ii) the location of pseudo-absences, and iii) the random allocation of an outbreak with one of the clades present a closed space-time window. Therefore, the analyses were bootstrapped 30 times and averaged to estimate the mean effect of these different steps involving stochasticity. There was a fairly quick convergence of results, which explains the relatively limited number of bootstraps. The analyses were also run with different definition of the space-time windows linking outbreak to sequence data to check the sensitivity of results to this parameter.

## Additional Information

**How to cite this article**: Artois, J. *et al*. Clade-level Spatial Modelling of HPAI H5N1 Dynamics in the Mekong Region Reveals New Patterns and Associations with Agro-Ecological Factors. *Sci. Rep.* **6**, 30316; doi: 10.1038/srep30316 (2016).

## Supplementary Material

Supplementary Information

## Figures and Tables

**Table 1:** Count of sequence and outbreak data following the bootstrapped linkage procedure (mean number ± standard deviation) for the three selected clades: the clade 1, clade 2.3.2 and clade 2.3.4.

		2004–2006	2007–2009	2010–2013
Clade 1	Sequences	368	137	155
	Outbreaks	5540.5 ± 10.4	180.1 ± 2.5	80.7 ± 4
Clade 2.3.2	Sequences	13	24	370
	Outbreaks	76.8 ± 7.1	14.2 ± 2.1	182.7 ± 5.5
Clade 2.3.4	Sequences	30	266	45
	Outbreaks	107.4 ± 7.8	227.4 ± 3.7	22.6 ± 3.4

**Table 2:** Predictive performance of the bootstrapped general HPAI H5N1 suitability and individual clade models, quantified by their mean AUC ± standard deviation.

	AUC	2004–2006	2007–2009	2010–2013
Clade 1	Training	0.83 ± 0.02	0.95 ± 0.01	0.92 ± 0.03
	Validation	0.73 ± 0.01	0.90 ± 0.01	0.77 ± 0.04
Clade 2.3.2	Training	0.88 ± 0.04	0.98 ± 0.02	0.87 ± 0.03
	Validation	0.70 ± 0.02	0.82 ± 0.07	0.68 ± 0.03
Clade 2.3.4	Training	0.88 ± 0.04	0.95 ± 0.02	0.93 ± 0.03
	Validation	0.71 ± 0.02	0.88 ± 0.01	0.58 ± 0.07
