# Supplementary material for: Clade-level Spatial Modelling of HPAI H5N1 Dynamics in the Mekong Region Reveals New Patterns and Associations with Agro-Ecological Factors
Source: Sci Rep. 2016 Jul 25;6:30316. doi: 10.1038/srep30316 (PMC4958987; doi:10.1038/srep30316)
Supplement: Supplementary Information [file srep30316-s1.pdf]

Clade-level Spatial Modelling of HPAI H5N1 Dynamics in the Mekong Region Reveals New Patterns and Associations with Agro-Ecological Factors: Supplementary Information

Jean Artois, Scott H. Newman, Madhur S. Dhingra, Celia Chaiban, Catherine Linard, Giovanni Cattoli, Isabella Monne, Alice Fusaro, Ioannis Xenarios, Robin Engler, Robin Liechti, Dmitri Kuznetsov, Long Pham Thanh, Nguyen Tung, Dong Pham Van, David Castellan, Sophie Von Dobschuetz, Filip Claes, Gwenaëlle Dauphin, Ken Inui, Marius Gilbert

Table of Contents

Supplementary Table S1 2

Supplementary Text S2 3

Figure A. Partial dependence plots for the four top predictor variables of suitability models 4

Table A. Number of clade/sequence linked to outbreaks for different distance thresholds 5

Table B. Predictive performance of the bootstrapped suitability model quantified by their mean AUC ± standard deviation 5

Table C. Mean relative contribution of each predictor variable to the prediction of HPAI H5N1 presence 5

Supplementary Text S3 6

Supplementary Table S1. Count of outbreak data following the bootstrapped linkage procedure (mean number ± standard deviation)

	2004 - 2006	2007 - 2009	2010 - 2013
**Clade 0**	15.9 ± 3.4	0 ± 0	0 ± 0
**Clade 1**	5540.5 ± 10.4	180.1 ± 2.5	80.7 ± 4
**Clade 2.3.2**	76.8 ± 7.1	14.2 ± 2.1	182.7 ± 5.5
**Clade 2.3.4**	107.4 ± 7.8	227.4 ± 3.7	22.6 ± 3.4
**Clade 5**	35.4 ± 3.8	0 ± 0	0 ± 0
**Clade 7**	0 ± 0	5.3 ± 1.5	0 ± 0

Supplementary Text S2. Model sensitivity to the spatial window radius.

To assign clades to outbreaks, a spatial window was defined around each outbreak. The model sensitivities to spatial window radius were tested by repeating the analysis with various radius sizes and the results for the clade 2.3.4 and the period from 2010 to 2013 was presented further. Six radiuses were tested: 50km, 100km, 150km, 200km, 250km and 300km. The clade-level suitability models were computed again with the new setting parameters.

First, the number of clade/sequence linked to outbreaks for the different distance thresholds was summarised in Table A. The buffer drawn around each outbreak location could be empty (0 clade/sequence) or including a number of clade/sequence ranging from 1 to 4. A radius of 150km was considered as a good trade-off between the uncertainty (2-4) and data lost (0) during the linking procedure.

The AUC scores, the relative contribution of the different predictor variables and the dependency profiles of the predicted values are presented in Table B, Table C and Figure A respectively. These results were relatively homogeneous and no major shifts were noted.

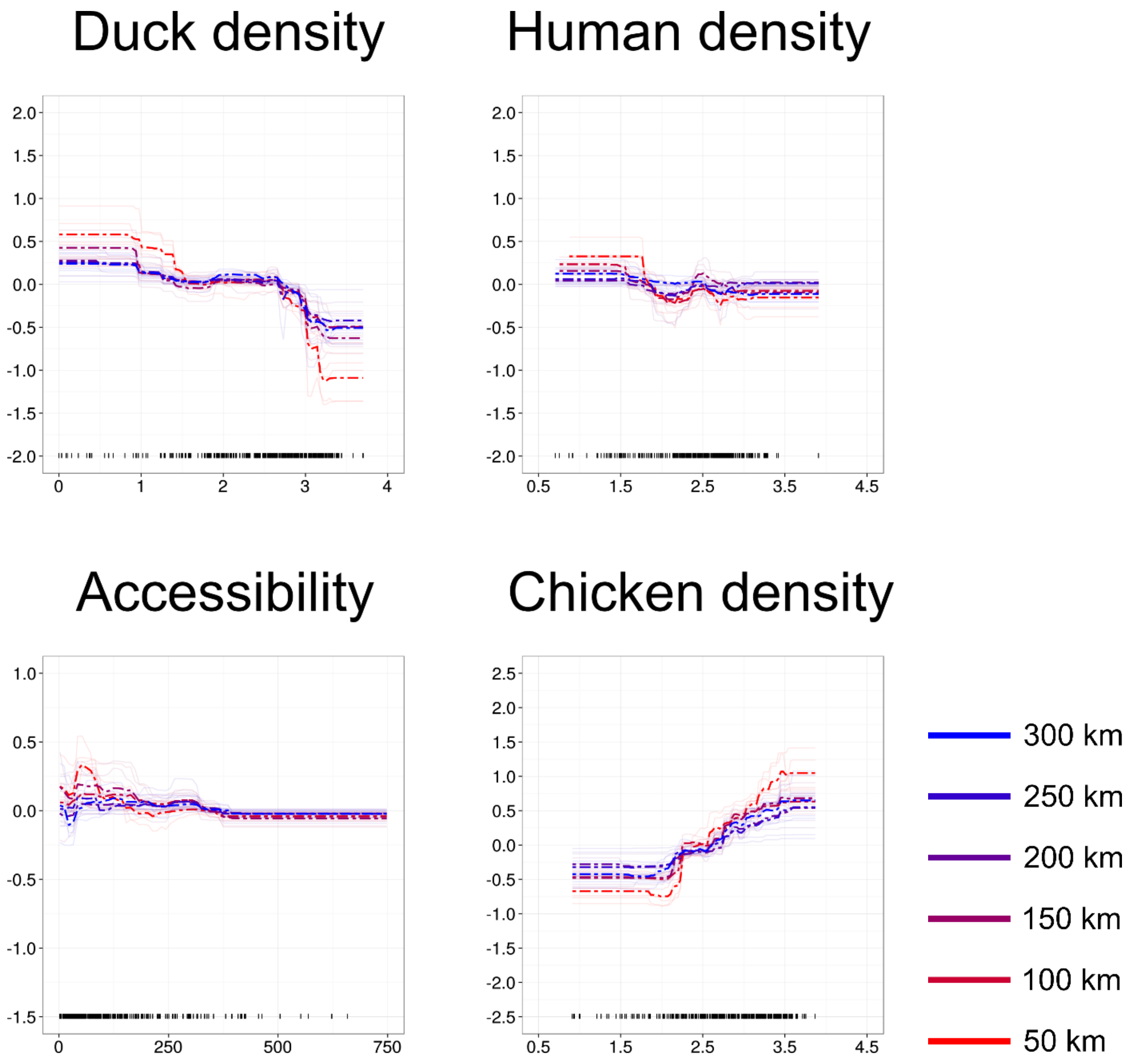

Figure A. Partial dependence plots for the four top predictor variables of suitability models (clade 2.3.2 from 2010 to 2013). The dashed line represents the mean profile, whilst transparent lines represent each bootstrap. The black ticks at the bottom of each plot represent the observed distribution of predictors for one bootstrap and the corresponding dataset. The colour gradient of these profile match to the six space windows used in the assignment procedure.

Table A. Number of clade/sequence linked to outbreaks for different distance thresholds.

	50 km	100 km	150 km	200 km	250 km	300 km
**0**	1310	502	338	209	110	64
**1**	4270	4169	3736	3560	3466	3398
**2**	1162	1609	2096	2316	2477	2548
**3**	81	539	639	713	738	769
**4**	14	4	14	25	32	44

Table B. Predictive performance of the bootstrapped suitability model quantified by their mean AUC ± standard deviation (clade 2.3.2 from 2010 to 2013).

	50 km	100 km	150 km	200 km	250 km	300 km
**Training**	0.90 ± 0.03	0.84 ± 0.01	0.86 ± 0.02	0.82 ± 0.02	0.83 ± 0.05	0.83 ± 0.03
**Validation**	0.69 ± 0.05	0.65 ± 0.03	0.66 ± 0.01	0.62 ± 0.03	0.61 ± 0.06	0.63 ± 0.02

Table C. Mean relative contribution of each predictor variable (± standard deviation) to the prediction of HPAI H5N1 presence (clade 2.3.2 from 2010 to 2013).

	Access	ChDnLg	DuDnLg	CropLandI	HpopDnLg	Wapc
**50 km**	14.43 ± 0.73	32.65 ± 2.56	31.68 ± 1.80	4.49 ± 3.30	16.03 ± 2.97	0.72 ± 0.43
**100 km**	11.75 ± 3.88	33.64 ± 5.46	27.64 ± 4.90	5.18 ± 3.33	21.3 ± 8.63	0.5 ± 0.36
**150 km**	13.71 ± 3.93	29.93 ± 4.41	28.93 ± 5.32	6.46 ± 3.29	20.91 ± 6.55	0.06 ± 0.05
**200 km**	10.57 ± 3.02	33.30 ± 3.66	32.16 ± 5.53	8.45 ± 3.56	15.31 ± 5.02	0.21 ± 0.23
**250 km**	13.78 ± 3.76	34.16 ± 8.23	34.33 ± 5.24	1.83 ± 0.93	15.86 ± 6.07	0.04 ± 0.09
**300 km**	14.56 ± 2.96	35.06 ± 2.04	31.03 ± 3.84	5.24 ± 5.13	13.67 ± 3.34	0.43 ± 0.45

24

Supplementary Text S3. Farm density modelling.

In addition to the chicken and duck density layers, we also aimed to produce a layer describing the epidemiological units, i.e. the number of farms per pixel, so that it could be used to spatialize presence and pseudo-absence points in a more realistic way than by random allocation. In Thailand and Viet Nam, the number of chicken and duck farms was available at the sub-district and commune level, respectively. We used these data sets to create farm distribution models, and the number of farms per administrative unit was modelled as a function of the number of chickens or ducks in the unit (chicken for the chicken farm model, ducks for the duck farm model), the human population, and the size of the administrative unit. Four models were computed: a chicken farm and a duck farm model were constructed separately for Thailand and Viet Nam, as these countries have a distinct agro-environment [1]. The model trained at the administrative level was then reapplied at the pixel level to derive the number of chicken and duck farms per pixel respectively. For Laos and Cambodia, the Viet Nam model was applied (with predictors from these countries) because little data on farm distribution was available from these countries, with poultry farming systems being closer to production found in Viet Nam than from typically more intensified production found Thailand [1]. Boosted Regression Tree models (BRT) were employed to model farm numbers as a function of these predictors.

For Thailand, the farm distribution models showed good performances, with correlation coefficients of 0.917 and 0.935 between the observed and modelled log-transformed numbers of ducks and chicken farms, respectively. When quantified on a validation set, these figures were reduced to 0.909 and 0.928. In Viet Nam, a similar model yielded correlation coefficients of 0.901 and 0.953 with the model set, and of 0.896 and 0.948 with the validation set for duck and chicken farms, respectively. One

can note a slightly better fit of the chicken farm models compared to the duck farms models.

1. Rushton J, Viscarra R, Guernebleich E, Mcleod A. Impact of avian influenza outbreaks in the poultry sectors of five South East Asian countries (Cambodia, Indonesia,, Lao PDR, Thailand, Viet Nam) outbreak costs, responses and potential long term control. Proceedings of the Nutrition Society. 1 sept 2005;61(3):491-514. doi: 10.1079/WPS200570.
